# Adrenal pheochromocytoma presenting with Takotsubo-pattern cardiomyopathy and acute heart failure: A case report and literature review

**DOI:** 10.1097/MD.0000000000004846

**Published:** 2016-09-09

**Authors:** Yi-Lun Chiang, Pei-Chi Chen, Chin-Cheng Lee, Su-Kiat Chua

**Affiliations:** a Division of Endocrinology; b Division of Pathology; c Division of Cardiology, Department of Internal Medicine, Shin Kong Wu Ho-Su Memorial Hospital, Taipei, Taiwan.

**Keywords:** blood volume, heart failure, pheochromocytoma, Takotsubo cardiomyopathy

## Abstract

**Background:**

Pheochromocytoma is an endocrine tumor that causes hypertension, facial pallor, and headache. Pheochromocytoma patients rarely present with acute heart failure or cardiogenic shock.

**Method:**

We discuss the case of a female patient with Takotsubo-pattern cardiomyopathy who presented with acute heart failure caused by pheochromocytoma.

**Result:**

Treatment was adjusted based on the data of the pulse contour cardiac output system. After intensive hydration and medication for heart failure, the condition of the patient stabilized.

**Conclusion:**

Before confirming the diagnosis, pulse contour cardiac output data could provide a direction for diagnosis and treatment.

## 1 Introduction

Pheochromocytoma is a rare endocrine tumor that secretes catecholamine and presents with a classic triad of headache, sweating, and palpitation.^[1]^ Pheochromocytoma-induced cardiomyopathy has been found to be similar to Takotsubo cardiomyopathy^[2]^ and myocarditis. Therefore, confirming the diagnosis before laboratory data are available is a challenge.

## 2 Case

**Figure 1:**
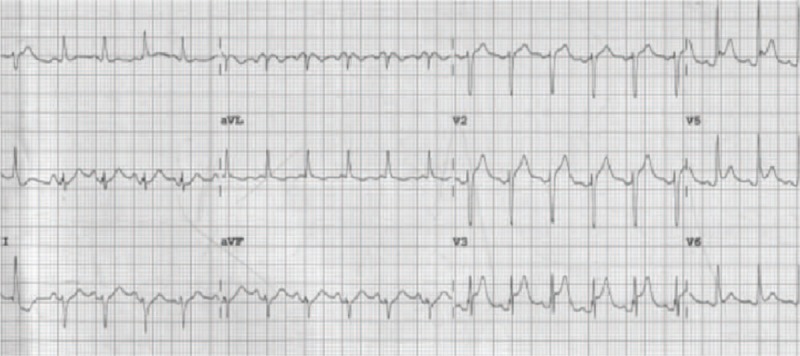
Electrocardiogram obtained on admission showing the sinus tachycardia with ST-segment elevation at V1 through V4.

**Figure 2:**
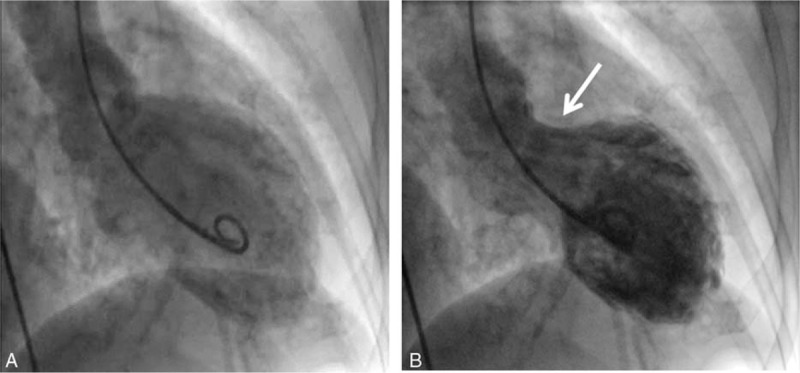
Left ventriculogram obtained on the first day of admission. Discriminating motion of the left ventricle is shown. Akinesis of the apical segment of the left ventricle, and excessive basal and middle motions (arrows) are apparent. A, Right anterior oblique (RAO) view during diastole; B, RAO during systole.

A 70-year-old woman presented to the emergency department with chest tightness associated with severe dyspnea, nausea, and vomiting. A review of her history revealed that she had intermittent episodic headaches for 1 year. At presentation, her pulse was 110 beats/min; blood pressure (BP) 154/96 mm Hg; body temperature (BT) 36°C; respiratory rate (RR) 30 breaths/min; and oxygen saturation 98% in 3 L of oxygen administered through a nasal cannula (Table 1). Electrocardiography revealed sinus tachycardia with ST-elevation at V_1_ through V_4_ (Fig. 1). A chest film image showed a borderline heart size and bilateral increased basal lung infiltration. Under the impression of ST-elevation myocardial infarction, the patient underwent emergent cardiac catheterization, which revealed no appreciable coronary artery stenosis. However, left ventricular angiography revealed akinesis of the apical segments of the left ventricle (LV), and excessive basal and middle contractions (Fig. 2). The LV end-diastolic pressure was 20 mm Hg.

**Table 1:**
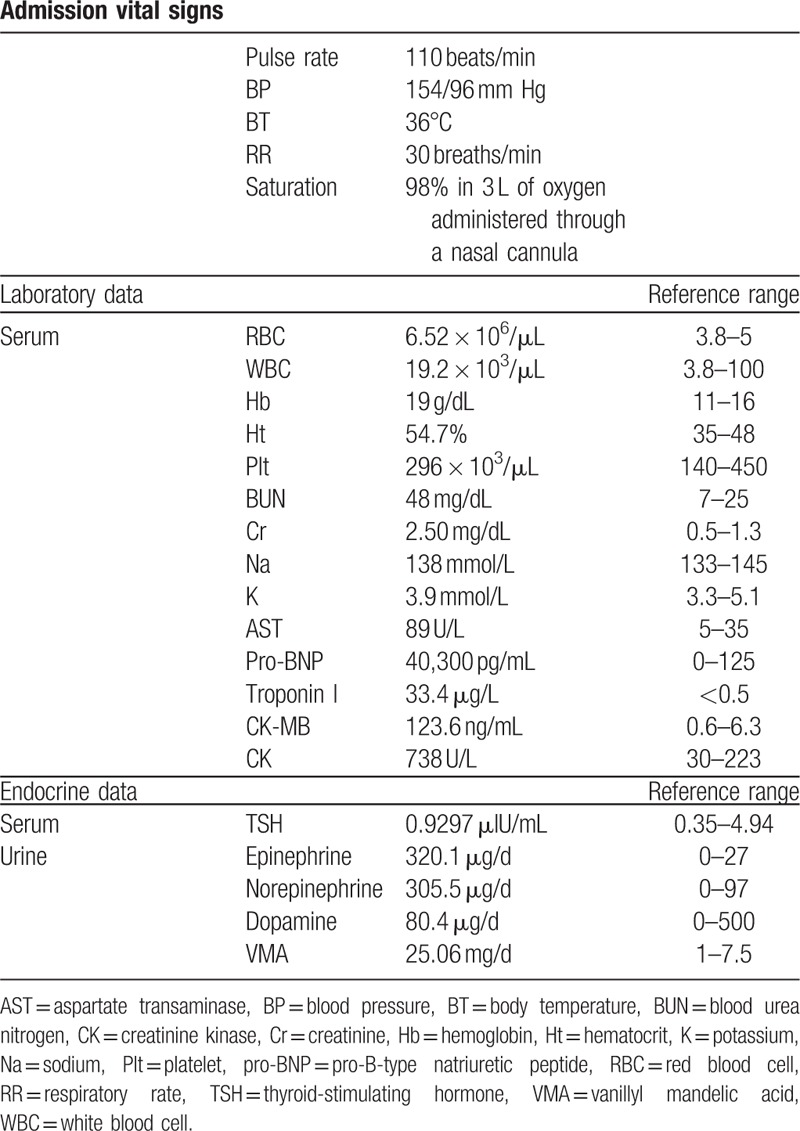
The patient's profile.

The plasma concentration of the pro-B-type natriuretic peptide (BNP) was 40,300 pg/mL. Other data included the following values: troponin I, 33.4 μg/L (<0.5 μg/L); creatinine kinase (CK), 738 U/L (30–223 U/L); and CK-MB, 123.6 ng/mL (0.6–6.3 ng/mL).

Four hours later, her BP suddenly decreased to 72/50 mm Hg, and she developed acute shortness of breath, diaphoresis, and a cold appearance. Endotracheal intubation was performed because of respiratory failure, and profound shock was observed. The pulse contour cardiac output (PICCO) system (PULSION Medical Systems AG, Germany) was used to evaluate her hemodynamic condition. Evaluation revealed a cardiac index (CI) of 1.42 L/min/m^2^, a systemic vascular resistance index (SVRI) of 5177 dyn.sec/cm^5^.m^2^, an intrathoracic blood volume index (ITBVI) of 812 mL/m^2^ (850–1000 mL/m^2^), and a global end-diastolic volume index (GEDVI) of 650 mL/m^2^ (680–800 mL/m^2^). Echocardiography revealed apical akinesis with relatively preserved LV basal wall motion, and the LV ejection fraction (LVEF) was 11%.

Owing to the decreased ITBVI and CI, intensive hydration with normal saline and inotropic agents was prescribed. Antibiotics and steroids were administered because of the possibility of myocarditis. After the treatment, the PICCO data showed a CI of 3.69 L/min/m^2^, an SVRI of 1929 dyn.sec/cm^5^.m^2^, and an ITBVI of 701 mL/m^2^. Echocardiography revealed a reduction in abnormal contraction in the apical wall of the LV, and the LVEF was 64%.

On the fifth hospital day, the urine catecholamine levels (in samples collected on the first hospital day) revealed the following values: epinephrine, 320.1 μg/d (0–27 μg/d); norepinephrine, 305.5 μg/d (0–97 μg/d); dopamine, 80.4 μg/d (0–500 μg/d); and vanillyl mandelic acid (VMA), 25.06 mg/d (1–7.5 mg/d). The alpha blocker was prescribed under the suspicion of adrenal pheochromocytoma. Later, the patient was transferred to the ordinary ward in a relatively stable condition.

**Figure 3:**
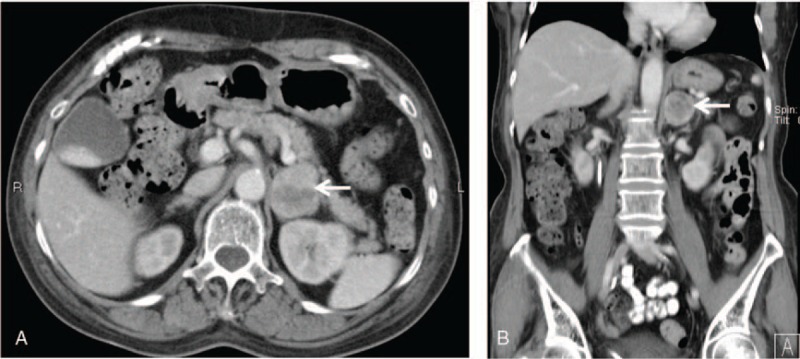
Abdominal computed tomographic scan showing a 3.9 × 3.4 × 3.0-cm well-defined heterogeneous enhanced tumor located in the left adrenal glands.

**Figure 4:**
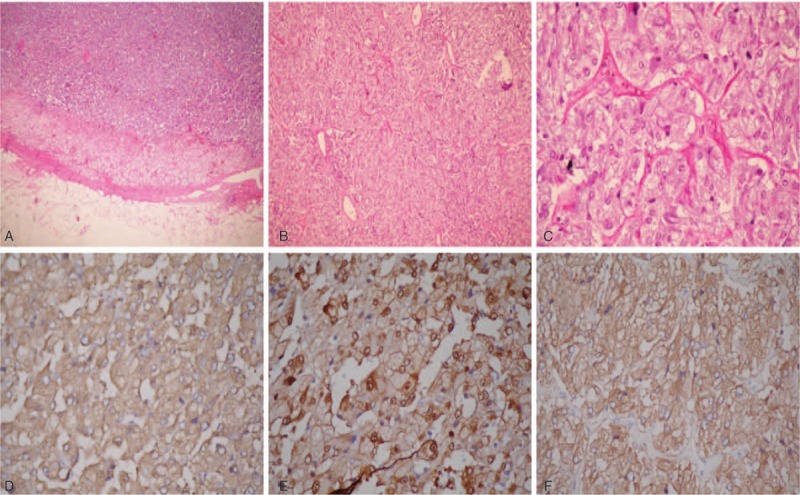
Macroscopic and microscopic appearance of the adrenal tumor. A, Normal adrenal and tumor cortex (∗); B, tumor cells in a nested arrangement and surrounded by fibrovascular stoma; C, tumor cells with basophilic glandular cytoplasm without atypia; D, chromogranin A positivity; E, S-100 positivity; F, synaptophysin positivity. Chromogranin A and synaptophysin are neuroendocrine markers, and S-100 is a marker of neural tissue.

A contrast-enhanced computed tomography scan of the abdomen showed a left adrenal heterogeneous enhanced mass measuring 3.9 × 3.4 cm (Fig. 3). The patient underwent an uncomplicated adrenalectomy, and the pathological report confirmed a pheochromocytoma (Fig. 4). The urine VMA was 3.7 mg/dL, and the dosage of the hypertension medication was tapered gradually. Patient informed consent was obtained.

## 3 Discussion

We present the case of an old woman with acute LV systolic dysfunction with no occluded coronary arteries. Initial differential diagnosis included Takotsubo cardiomyopathy, myocarditis, and pheochromocytoma with acute heart failure. Owing to the similar presentations of these conditions, diagnosis was difficult.

Pheochromocytoma is a rare neuroendocrine tumor, typically located unilaterally in the adrenal gland, which may release catecholamine products. Previous studies showed the incidence to be 0.05% to 0.8%.^[3]^ Owing to the secretion of catecholamines, patients typically present with a classic triad of headache, palpitation, and sweating. Clinical expression may involve numerous cardiovascular manifestations because of excess catecholamines, but typically present as hypertension.^[4]^

Pheochromocytoma with acute LV dysfunction is a stress-related cardiomyopathy.^[5]^ A literature review revealed cases of pheochromocytoma with the presentation of Takotsubo-pattern cardiomyopathy confirmed through ventriculography or echocardiography.^[2,6,7]^ The patients were relatively young. The clinical features varied from palpitation, dyspnea, and abdominal pain to shock and collapse. The electrocardiographic patterns have been heterogeneous, ranging from sinus tachycardia and ST change to bundle branch block. The patients received inotropic agents, intra-aortic balloon pumps, and even extracorporeal life support. Most patients survived if a suitable treatment was provided and a correct diagnosis made.

Takotsubo cardiomyopathy is characterized by a transient hypokinesis of the LV apex, and is typically induced by stressful emotional or physical events.^[8]^ Patients with Takotsubo cardiomyopathy tend to be older and are more commonly women.^[8,9]^ The clinical course resembles that of acute coronary syndrome or acute decompensated heart failure, and resolves within days to weeks. The prognosis is generally favorable.

Patients with myocarditis may have a viral prodrome, and various clinical scenarios can occur, such as acute coronary syndrome, acute dilated cardiomyopathy, and heart failure.^[10]^ Male patients are slightly more common than female patients,^[10]^ and a previous study revealed that the inflammation-related cardiac diastolic dysfunction was associated with plasma levels of tumor necrosis factor (TNF)-α and interleukin (IL)-6.^[11]^ These inflammatory factors down-regulated the expression of the sarcoplasmic reticulum Ca^2+^-ATPase gene and delayed diastolic calcium reuptake. Whereas the diastolic function improved, the levels of these cytokines decreased.^[11]^ Among the Chinese population, risk-conferring genetic variants of the angiotensin II type 1 receptor gene for diastolic heart failure were found, which were previously identified.^[12]^

Based on clinical presentation alone, diagnosis is difficult to make, and collection of urine data of catecholamine and VMA takes time. Before the urinalysis results become available, PICCO data could provide an indication of the diagnosis. The hemodynamic status showed low CI and high SVRI among the 3 conditions. However, the intravascular volumes of Takotsubo cardiomyopathy and myocarditis were increased, whereas the intravascular volume of pheochromocytoma with acute heart failure was depleted. The low intravascular volume was attributable to the catecholamine-induced volume contraction. Therefore, volume expansion should be performed with caution in patients with pheochromocytoma. Hence, PICCO data could provide a direction for diagnosis and treatment before the data of urine VMA and catecholamine become available.

Our case indicated that a diagnosis of pheochromocytoma should be considered when echocardiography or angiography reveals Takotsubo-pattern cardiomyopathy. Moreover, PICCO data should be evaluated when patients have acute LV systolic dysfunction with heart failure and no coronary artery stenosis. Finally, for patients presenting with acute heart failure, pheochromocytoma should always be considered.
